# XTOP: high-resolution X-ray diffraction and imaging

**DOI:** 10.1107/S160057671500895X

**Published:** 2015-05-31

**Authors:** Vincent Favre-Nicolin, José Baruchel, Hubert Renevier, Joël Eymery, András Borbély

**Affiliations:** a Université Grenoble Alpes, INAC-SP2M, F-38000 Grenoble-Alpes, France; b CEA, INAC-SP2M, F-38000 Grenoble, France; c ESRF, 71 avenue des Martyrs, CS 40220, 38043 Grenoble Cedex 9, France; d Laboratoire des Matériaux et du Génie Physique, Grenoble INP – Minatec, Grenoble, France; e École Nationale Supérieure des Mines, SMS-EMSE, CNRS:UMR 5307, LGF, Saint-Étienne, France

**Keywords:** high-resolution X-ray diffraction, imaging, editorial

## Abstract

The latest virtual special issue of *Journal of Applied Crystallography* includes some highlights of the 12th Conference on High-Resolution X-ray Diffraction and Imaging (XTOP), which took place in Villard-de-Lans and Grenoble in September 2014.

The latest virtual special issue of *Journal of Applied Crystallography* includes some highlights of the 12th Conference on High-Resolution X-ray Diffraction and Imaging (XTOP). This biennial conference was held in Villard-de-Lans and Grenoble in September 2014, and was organized by the ESRF, Université Grenoble-Alpes, the CEA and CNRS. A refresher school for PhD students and postdoctoral scientists took place on the first day, with lectures on new diffraction and imaging techniques. 

The first XTOP conference (‘X-ray Topography and High Resolution Diffraction’) took place in Marseille in 1992. The original topics covered were diffraction topography, double- and triple-crystal diffractometry of epitaxial layers, reflectometry, and the standing wave technique. Many experimental techniques have been added since, which cover today a wide range to reflect the scientific evolution of the XTOP community: coherent imaging, X-ray phase contrast imaging (radiography and microtomography), microdiffraction (monochromatic and Laue), grazing incidence and resonant diffraction, fast (time-resolved) tomography *etc.* The topics covered now include both synchrotron and laboratory instrumentation, including industrial developments.

The materials studied include single crystals and multilayers, nanostructures (metals, oxides, semiconductors), and organic, biological and other natural (sea shells, fossils *etc.*) samples. A few presentations in 2014 covered advances in the theory of high-resolution X-ray diffraction, and with the occasion of the International Year of Crystallography, André Authier gave a presentation on the *Early Days of X-ray Diffraction and the Birth of the Dynamical Theory*. Among the topics presented during the XTOP conference, the most noteworthy included the development of high temporal and spatial resolution for diffraction and imaging, the use of X-ray coherent beams (including ptychography and X-ray free electron lasers), and applications to nanostructured materials.

Following the traditional publication policy of the XTOP conferences, the articles presented in this issue are not selected proceedings but rather original research articles, which were first presented at the XTOP conference. These include laboratory and synchrotron studies of semiconductors, software for fast data analysis or instrumental function simulation, micro-Laue analysis of oxide fuel cells and bent nanowires, microtomography of bone structure, X-ray phase contrast imaging of bacterial endospores, and Bragg imaging of ice under applied stress.

Over 200 participants attended the five-day conference, which included six tutorial lectures, 13 invited and 44 contributed presentations, and 144 posters.

